# Extracellular lipid loading augments hypoxic paracrine signaling and promotes glioma angiogenesis and macrophage infiltration

**DOI:** 10.1186/s13046-019-1228-6

**Published:** 2019-06-07

**Authors:** Svenja Offer, Julien A. Menard, Julio Enríquez Pérez, Kelin G. de Oliveira, Vineesh Indira Chandran, Maria C. Johansson, Anna Bång-Rudenstam, Peter Siesjö, Anna Ebbesson, Ingrid Hedenfalk, Pia C. Sundgren, Anna Darabi, Mattias Belting

**Affiliations:** 1 Department of Clinical Sciences Lund, Section of Oncology and Pathology, Lund University, Barngatan 4, SE-221 85 Lund, Sweden; 2 Center for Translational Cancer Research, Technical University of Munich, Munich, Germany; 3 Department of Clinical Sciences Lund, Section of Neurosurgery, Lund University, Lund, Sweden; 4 Department of Neurosurgery, Skåne University Hospital, Lund, Sweden; 5 Department of Clinical Sciences, Lund, Section of Diagnostic Radiology, Lund University, Lund, Sweden; 6 Department of Medical Imaging and Function, Skåne University Hospital, Lund, Sweden; 7 Department of Hematology, Oncology and Radiophysics, Skåne University Hospital, Lund, Sweden

**Keywords:** Glioma, Lipid metabolism, Hypoxia, Angiogenesis, Macrophages

## Abstract

**Background:**

Primary brain tumors, in particular glioblastoma (GBM), remain among the most challenging cancers. Like most malignant tumors, GBM is characterized by hypoxic stress that triggers paracrine, adaptive responses, such as angiogenesis and macrophage recruitment, rescuing cancer cells from metabolic catastrophe and conventional oncological treatments. The unmet need of strategies to efficiently target tumor “stressness” represents a strong clinical motivation to better understand the underlying mechanisms of stress adaptation. Here, we have investigated how lipid loading may be involved in the paracrine crosstalk between cancer cells and the stromal compartment of the hypoxic tumor microenvironment.

**Methods:**

Regions from patient GBM tumors with or without the lipid loaded phenotype were isolated by laser capture microdissection and subjected to comparative gene expression analysis in parallel with cultured GBM cells with or without lipid loading. The potential involvement of extracellular lipids in the paracrine crosstalk with stromal cells was studied by immunoprofiling of the secretome and functional studies in vitro as well as in various orthotopic GBM mouse models, including hyperlipidemic ApoE−/− mice. Statistical analyses of quantitative experimental methodologies were performed using unpaired Student’s T test. For survival analyses of mouse experiments, log-rank test was used, whereas Kaplan-Meier was performed to analyze patient survival.

**Results:**

We show that the lipid loaded niche of GBM patient tumors exhibits an amplified hypoxic response and that the acquisition of extracellular lipids by GBM cells can reinforce paracrine activation of stromal cells and immune cells. At the functional level, we show that lipid loading augments the secretion of e.g. VEGF and HGF, and may potentiate the cross-activation of endothelial cells and macrophages. In line with these data, in vivo studies suggest that combined local tumor lipid loading and systemic hyperlipidemia of ApoE−/− mice receiving a high fat diet induces tumor vascularization and macrophage recruitment, and was shown to significantly decrease animal survival.

**Conclusions:**

Together, these data identify extracellular lipid loading as a potentially targetable modulator of the paracrine adaptive response in the hypoxic tumor niche and suggest the contribution of the distinct lipid loaded phenotype in shaping the glioma microenvironment.

**Electronic supplementary material:**

The online version of this article (10.1186/s13046-019-1228-6) contains supplementary material, which is available to authorized users.

## Background

High-grade glial tumors (glioblastoma, GBM) are the most common and aggressive primary brain tumors in adults with a poor median overall survival of less than 1 ½ years [1, 2]. GBM, like most other malignant tumors, is characterized by hypoxic stress and extracellular acidification that trigger adaptive responses rescuing cancer cells from metabolic catastrophe and the cytotoxic effect of conventional oncological treatments with cytostatic drugs and ionizing radiation [3, 4]. Oncogenic signaling and GBM stressness have recently been linked to the dependence on lipids rather than glucose as a primary substrate for energy production [5, 6]. MR imaging studies in patient tumors reported that elevated lipid resonances are a characteristic of hypoxic tumor areas [7], and stressed cancer cells have shown increased de novo lipogenesis as well as extracellular lipid uptake ultimately resulting in lipid droplet (LD) formation and storage [4, 8–11].

Extracellular lipids and their storage into LDs thus emerges as an important, stress-adaptive mechanism in malignant tumors, and recent studies suggest that cancer cells assemble LDs to oxidize fatty acids for increased reductive potential and to rescue cells from oxidative stress [12, 13]. These studies have revealed an intrinsic role of LDs in energy homeostasis and scavenging of reactive oxygen species in cancer cells. However, the development and progression of malignant tumors depend on paracrine mechanisms to create a hypervascularized and immunosuppressive environment by e.g. increased angiogenesis and macrophage infiltration. Although an association between the abundance of LDs in cancer cells and tumor aggressiveness has been reported for several tumor types, including GBM [14] and prostate cancer [15], the underlying mechanisms of how extracellular lipids and LD storage may contribute to hypoxic signaling and activation of stromal cells to shape the stressed tumor niche remain unknown.

Here, we were interested in investigating how the LD phenotype at the mechanistic and functional level may be involved as a driver of the paracrine crosstalk between cancer cells and the stromal compartment of the hypoxic microenvironment of gliomas.

## Methods

### Clinical samples

Tumor biopsy specimens were obtained from patients with GBM (WHO grade IV) at the Department of Neurosurgery, Skåne University Hospital, Lund, Sweden. Tumor samples were collected with informed consent according to Protocol H15642/2008 approved by the Lund University Regional Ethics Board, Sweden. Patient plasma samples were from a population-based trial cohort (“MRI study”) encompassing patients referred to the Neurosurgery Department at Lund University Hospital, Lund, with a suspicion of an intracranial tumor [16]. The study was carried out according to the ICH/GCP guidelines and in agreement with the Helsinki declaration, and was approved by the local ethics committee, Lund University (Dnr.2011/814, and 2012/188). Patients were diagnosed by routine MRI of the brain, surgical and pathological procedures, received standard oncological treatment and were followed up according to national recommendations. Blood samples from patients at baseline (pre-operative) and healthy control donors were collected in EDTA tubes, centrifuged at 2000 x g for 10 min at room temperature (RT) and plasma was stored at − 80 °C.

### Animal studies

Experimental procedures were approved by the ethical committee for animal research in Malmö/Lund (ethical permit M144–14 and M145–14) and were performed in accordance with the European Union animal rights and ethics directives. Apolipoprotein E knock out (ApoE^−/−^) mice were fed with high fat diet (21% fat) (R638, Lantmännen, Sweden) starting 4 weeks prior to cell injection. To obtain GL261 mouse glioma cells with and without LDs, cells were seeded in DMEM containing 10% FBS overnight (O/N), washed twice in PBS and incubated in hypoxia in serum-free (SF) medium in the presence or absence of low density lipoprotein (LDL, 50 μg/mL) for 48 h. Cells were kept subconfluent, detached in trypsin-EDTA, and allowed to recover in DMEM with 10% FBS for 20 min during counting/viability measurements. The cells were then washed twice and finally resuspended in SF DMEM without antibiotics. For orthotopic injections, 8–12 weeks old NOD/SCID (Taconic Biosciences), C57BL/6, or ApoE^−/−^ mice (The Jackson Laboratory, JAX) were anaesthetized with isoflurane and placed on a stereotactic frame. Mice received Visco-tears and local anesthetic *s.c.* (Marcaine), a hole was drilled in the skull and 5 μL of the cell suspension (50.000 or 100.000 cells, as indicated in the respective figure legend) was slowly injected over 2–3 min 1.5 mm to the right and 1.0 mm anterior of bregma, 2.5 mm deep from the dural surface using a Hamilton syringe. The needle was left in place for 2 min, and slowly retracted before the skull hole was filled with bone wax and the wound closed with clips. Mice finally received Temgesic analgesic (0.3 mg/mL) *s.c.* in the posterior leg. Mice were monitored daily and sacrificed at the indicated time-points or whenever displaying symptoms of distress. The mice brains were then collected and snap-frozen in isopentane before storage at − 80 °C and sectioning for histological analyses.

### Cell-lines

GBM U-87 MG (newly purchased from ATCC) and GL261 cells were routinely cultured in high-glucose DMEM (HyClone, GE) medium supplemented with 10% (v/v) FBS (Sigma Aldrich, F7524), 2 mmol/L L-glutamine (Sigma Aldrich, G7513), 100 U/ml penicillin, and 100 μg/ml streptomycin (PEST; Sigma Aldrich, P0781). HBMECs were cultured in EC medium (3H Biomedical) supplemented with 5% (v/v) FBS, 1% (v/v) EC growth supplement and 1% (v/v) PEST. HBMECs at passages 2–5 were used for experiments. THP-1 cells were maintained in RPMI 1640 medium (HyClone) containing 10% (v/v) FBS, 2 mmol/L L-glutamine and 1% (v/v) PEST. The differentiation of THP-1 cells to a macrophage-like state was induced by adding 50 nM phorbol-12-myristate-13-acetate (PMA, Sigma Aldrich) for 48 h, followed by 48 h recovery in SF medium in the absence of PMA. All cells were grown in a humidified 5% CO_2_ incubator at 37 °C. For hypoxia experiments, cells were incubated in a humidified Sci-tive-NN-Hypoxia workstation (Ruskinn Technology) set at 5% CO_2_, 94% N_2_, 1% O_2_, and 37 °C. All hypoxia experiments were performed at SF conditions unless stated otherwise.

### Laser microdissection and gene expression

Human GBM tumor cryosections (10 μm) were mounted onto DNase and RNase free membranes (FrameSlide PET), rapidly stained for nuclei using Cresyl Violet (Ambion) and dehydrated in ice-cold ethanol. Slides were stored in sterile 50 ml tubes at -80 °C before Laser Capture Microdissection (LCM) using the PALM system from Zeiss. To identify areas of interest, adjacent sections were mounted onto poly-lysine coated slides, fixed with 70% (v/v) ethanol for 1 min, stained for nuclei (DAPI) and LDs using HCS LipidToxTM green neutral lipid stain (Life Technologies). Dissected, LD-loaded and non-LD-loaded tumor areas (*N* = 5 patients with a total area of approximately 10 mm^2^) were dissolved in 50 μl lysis solution provided with AllPrep DNA/RNA Mini Kit (Qiagen), and RNA isolation was performed according to the manufacturer’s instructions. RNA concentration and purity were determined with the BioAnalyzer, and quantified using the Affymetrix Clariom D Pico Gene Array at the BEA core facility, Karolinska Institute, Stockholm. Background correction, normalization and annotation were performed with R statistical language within Rstudio environment (www.r-project.org and https://www.rstudio.com/) with the packages affycoretools, oligo, clariomdhumanprobeset.db and pd.clariom.d.human, all available in the Bioconductor repository (https://www.bioconductor.org/). Further analyses were performed on genes considered as differentially upregulated (cutoff of 0.5 log_2_FC) for TC^LD+^ vs TC^LD-^ comparison also in R.

### Gene expression array and data processing

U-87 MG cells were grown in SF DMEM for 48 h in normoxia (N), hypoxia (H), or H + LDL (AlfaAesar/ThermoFisher, 50 μg/mL). For cell reoxygenation experiments, cells were grown for 48 h H or H + LDL (50 μg/mL) followed by 1 M NaCl wash, two PBS washes and two DMEM washes (to remove remaining LDL in cell culture), before another incubation period of 6 h or 48 h reoxygenation in N (20% O_2_) in SF DMEM. U-87 MG NA and AA cells were grown in SF DMEM for 48 h in pH 7.4 or 6.7. Independent, triplicate experiments were performed for all conditions. Total RNA was extracted using RNAeasy mini kit (Qiagen). Further processing and QC by Bioanalyzer were performed at the SCIBLU Genomics Centre at Lund University for hybridization on HumanHT-12v4 Expression Illumina BeadChip. Downstream background correction, normalization, annotation and differential expression analysis were performed with R statistical programming language within Rstudio environment, using limma package, available at the Bioconductor repository. Subsequent level 4 GO annotation for cellular component and biological processes analyses were performed with Over-representation Analysis tool available at ConsensusPathDB-Human (http://cpdb.molgen.mpg.de), with *p*-value cutoffs at 0.01. Plots on the scrutiny of differentially expressed genes (*p*-value< 0.01) and on pathway analysis results were made with graphical packages for R environment, such as pheatmap, RColorBrewer and ggplot2, all available at CRAN repository (https://cran.r-project.org/web/packages/).

### Multiplex proximity extension assay and ELISA

U-87 MG cells were treated at the different conditions as indicated above for gene array analyses. Conditioned medium (CM) was collected and spun twice at 300×g for 5 min to remove cell debris, freeze-dried and resuspended in PBS before BCA protein quantification. Secreted proteins in CM as well as in GBM patient and healthy control plasma were analyzed using the Proseek Multiplex Oncology II^96x96^ and CVD III^96x96^ panels (Olink Bioscience), as previously described [16–18]. Briefly, the protein quantification is based on proximity extension assay (PEA) technology, which provides high sensitivity and specificity based on the binding of oligonucleotide-labelled antibody probe pairs to their specific target protein, generating a PCR-amplified DNA template, which is proportional to the initial antigen concentration as quantified by real-time qPCR. Four internal and three negative controls were used to calculate the lower limit of detection for each protein. For each analyte/protein detected, difference of normalized protein expression (NPX, log2) between samples was calculated, and linear fold change was obtained with the formula: linear fold change = 2^NPX^.

Validation of some identified proteins (VEGF-A and HGF) was performed using human HGF and VEGF-A Quantikine ELISA assays (R&D systems) according to manufacturer’s instructions. The absorbance was measured at 450 nm and 540 nm in a spectrophotometer (FLUOstarOPTIMA).

### Western blotting

Cells were lysed in RIPA buffer (10 mM Tris-HCl pH 7.4, 150 mM NaCl, 1 mM EDTA, 0.1% SDS, 1% Triton X-100, 1% sodium deoxycholate) supplemented with Complete protease inhibitor (Roche Diagnostics). Lysates were centrifuged at 18000×*g* for 10 min, and supernatants were stored in -20 °C prior to analysis. Protein concentration was measured by BCA assay. For analysis of CM from U-87 MG cells, debris was removed by centrifugation, and supernatants were freeze-dried and resuspended in RIPA buffer with Complete protease inhibitor for analysis. Equal amounts of total protein (or equal volumes for CM samples) were mixed with LDS sample buffer and reducing agent and separated by electrophoresis in a 4–12% NuPAGE Bis-Tris gel with SeeBlue Plus2 (Invitrogen) as molecular mass standard. The proteins were electroblotted to PVDF membranes (Immobilon-FL, Merck Millipore). After blocking in 5% nonfat milk in TBS-Tween20 (0.1%) buffer (blocking buffer) for 1 h at RT, membranes were probed with the indicated primary antibody (anti-CA9 1:400 (BioScience Slovakia, M75)), anti-vimentin 1:2000 (GeneTex; GTX100619), anti-HIF1α 1:1000 (GeneTex, GTX127309), anti-HIF2α 1:500 (Abcam, Ab199)) O/N at 4 °C in blocking buffer. The membrane was washed in TBS-Tween20 and incubated with HRP-conjugated secondary antibody for 1 h, at RT. Signal detection was performed using ECL Western blotting substrate (Pierce) and quantification of the bands by densitometry using ImageJ (NIH).

### Phosphokinase array

Cells (1 × 10^6^) were seeded in T-25 cm^2^ flasks, starved in SF DMEM medium O/N at N or H, and stimulated or not with LDL (50 μg/ml) for 5, 15, 30, 45, 60 or 90 min. Cells were lysed in RIPA buffer supplemented with Protease Inhibitor Mixture tablets and phosphatase inhibitors (PhosSTOP, Roche). Total protein from short treatments (5, 15, 30 min) and longer treatments (45, 60, and 90 min) were equally mixed and 300 μg was analyzed using the Human P-Kinase Array (R&D Systems) as indicated by the manufacturer. The spot intensity was analyzed using the Protein Array analyzer for ImageJ and fold change of relative protein levels (normalized to array reference) was calculated.

### Cell migration

HBMECs were starved in SF EC medium (1% L-Glut, 1% PEST) O/N. Cells (4 × 10^4^) were added in SF media to the top chamber of 8-μm pore cell culture inserts (BD Biosciences) placed in a 24-well plate. THP-1 cells were differentiated in the top chamber of 8-μm pore cell culture inserts for 48 h, followed by starvation in SF medium for 48 h. Cells were incubated at 37 °C for 6 h to allow cell migration toward SF medium, or different CM (diluted 1:5 with SF medium for HBMECS and undiluted for THP-1 cells) collected from U-87 MG and GL261 cells, as indicated in the respective figure legend. Migrated cells attached to bottom membrane were fixed with 4% paraformaldehyde (PFA) for 15 min, stained with crystal violet, and counted from pictures taken under the microscope (Axiovert 40C, 4× objective; Carl Zeiss).

### Matrigel tube formation

HBMECs were pre-treated with the different CM derived from U-87 MG cells for 24 h and were then seeded into a growth factor-reduced BD Matrigel (BD Bioscience) pre-coated 96-well plate at 2 × 10^4^ cells per well in EC medium supplemented with 5% (v/v) FBS and EC growth supplement. EC tube formation was captured 20 h after seeding (Axiovert 40C microscope, 4× objective; Carl Zeiss). The angiogenic property was assessed by counting the number of tubes per condition.

### Cell proliferation

HBMECS were seeded (1 × 10^3^ cells) in a 96-well plate and starved in SF medium O/N. Cells were incubated in SF medium or in different CM of U-87 MG or GL261 cells for a total of 72 h. During the last 3 h, cells were cultured in the presence of 20 μl/well MTS Reagent (Abcam). The quantification of metabolically active cells was performed by measuring the absorbance at 490 nm.

### Immunohistochemistry

Sections (6 μm) were prepared from mouse brains snap frozen in isopentane. H&E stainings were performed on a Leica ST4020 stainer on every 3rd adjacent sections for morphological analysis and for largest tumor diameter measurement. Pictures were captured with an Olympus BX53 microscope or MiraxMidi Zeiss slide scanner for whole slide images. Tumor areas from whole slide pictures were then measured with ImageJ.

### Immunofluorescence

Mouse brain tumor cryosections (6 μm) were washed/rehydrated in PBS for 5 min, fixed in 4% PFA and washed twice in PBS before blocking with 5% normal goat serum in PBS (blocking solution) for 1 h, at RT. Primary antibodies (F4/80, 1:10 (Biorad, CI:A3–1); αSMA, 1:100 (Abcam, Ab5694); vWF, 1:100 (Abcam, Ab11713) were then added in blocking solution and incubated O/N at 4 °C. Sections were washed, followed by incubation with secondary anti-IgG fluorescent antibodies AF594-donkey anti-rat, 1:200; AF488-goat anti-rabbit, 1:200; and AF594-donkey anti-sheep, 1:200, respectively, for 1 h, at RT. Secondary antibody alone was performed as controls. Nuclei were counter-stained with DAPI. Sections were washed and mounted with fluorescent mounting medium, and slides were observed on an Olympus BX53 fluorescence microscope and pictures captured for quantification. Fluorescence area was quantified using ImageJ on single channel pictures and normalized to the corresponding nuclei area of the same field. The number of fields used for quantification is indicated in the corresponding figure legends.

LD staining was performed as previously described [8]. Briefly, cells were washed in PBS and fixed with 4% PFA before staining with HCS Green LipidTox staining and Hoechst 33342, washed again in PBS and observed under Zeiss LSM710 confocal microscope using a Plan-Apochromat 63x/1.4 Oil DIC M27 objective.

### Statistical analyses

Statistical analyses of quantitative experimental methodologies were performed using unpaired Student’s T test with the GraphPad prism suite (La Jolla, CA, USA). Data is presented as means ± SD. For survival analyses of mouse experiments, log-rank test was used, whereas Kaplan Meier was performed to produce survival curves from the TCGA patient data. Statistical significance was set at α = 0.05 level unless otherwise indicated. Empirical Bayes statistics was used to calculate log-odds ratio, moderate f-statistics and moderate t-statistics for microarray data within R environment. Heatmaps were clustered according to Ward’s minimum variance method.

## Results

### Lipid loading associates with enhanced hypoxic paracrine signaling in patient GBM

**Fig. 1:**
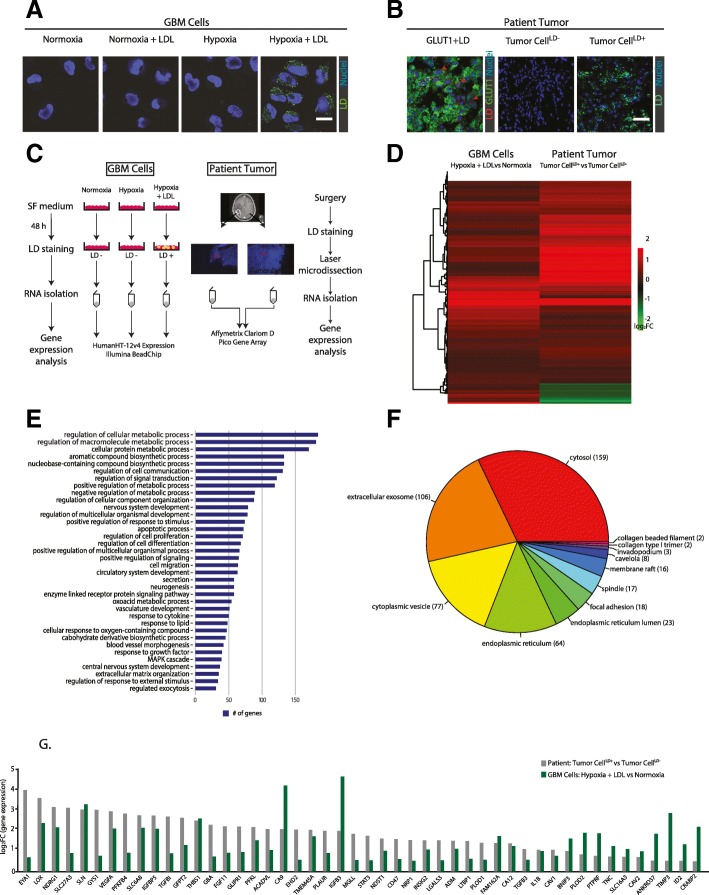
Comparative gene expression profiling of the LD^+^ phenotype in GBM patient tumors and cells. **a**, GBM cells (U-87 MG) were incubated in serum-free medium at normoxic and hypoxic (1% O_2_) conditions for 48 h without or with extracellular lipid (LDL, 50 μg/ml) and analyzed for LDs by confocal microscopy; blue, Hoechst nuclear stain; green: LipidTox LD stain. Shown are representative images of at least three independent experiments. Scale bar, 20 μm. **b**, Immunofluorescence images of GBM patient tumors. Left panel (GLUT1 + LD) shows co-localization of the hypoxia marker GLUT1 (green) and LDs (Nile red; indicated by red arrowheads). Mid (Tumor Cell^LD-^) and right (Tumor Cell^LD+^) panels show typical tumor areas with and without LDs (LipidTox, green). Blue, Hoechst nuclear stain. Scale bar, 100 μm. **c**, Schematic overview of the experimental set-up of gene expression analyses. Left: GBM cells (U-87 MG) were incubated in serum-free (SF) medium at normoxic and hypoxic (1% O_2_) conditions for 48 h with no addition of extracellular lipid or in hypoxia with extracellular lipid (LDL, 50 μg/ml), and subsequently analyzed for gene expression with the HumanHT-12v4 Expression Illumina BeadChip (*N* = 3 independent experiments). Right: GBM tumors (*N* = 5 patients) were cryosectioned, and LD-negative (Tumor Cell^LD-^) and LD-positive (Tumor Cell^LD+^) tumor areas (*N* = 8 and 10, respectively) were isolated by laser microdissection for gene expression analysis with Affymetrix Clariom D Pico gene array. **d**, Heatmap showing the differentially upregulated genes in LD^+^ (Hypoxia + LDL) vs LD^−^ (Normoxia) GBM cells and how the same set of genes are differentially expressed in Tumor Cell^LD+^ vs Tumor Cell^LD-^ areas from GBM patient tumors. **e**, Biological processes plot generated from the commonly upregulated genes shown in (**d**) according to Gene Ontology level 4 annotation, *P* < 0.01. **f**, Cellular component analysis generated as described in (**e**). **g**, Significantly upregulated genes of interest in Tumor Cell^LD+^ vs Tumor Cell^LD-^ from patient tumors (gray bars) and LD^+^ (Hypoxia + LDL) vs LD^−^ (Normoxia) GBM cells (green bars). Data in (**d**) and (**g**) are presented as log_2_ of fold-change (FC)

In agreement with previous studies [16], GBM cells grown in normoxia, hypoxia, or normoxia in the presence of extracellular lipid (+LDL) did not display lipid droplets (LDs), whereas hypoxic cells provided with LDL acquired the lipid loaded phenotype (Fig. 1a). Accordingly, GBM patient tumors showed areas with and without LDs, and LD-positive cells resided in the hypoxic tumor niche, as indicated by co-staining for the hypoxia marker GLUT-1 ((Fig. 1b). To gain a better understanding of the nature of GBM patient tumor cells with the lipid loaded phenotype, we comprehensively profiled tumor cell LD positive (TC^LD+^) and LD negative (TC^LD-^) regions captured from GBM tumors by laser capture microdissection (LCM). In parallel, TC^LD+^ and TC^LD-^ from human GBM in vitro cultures were profiled for comparative gene array analysis using the most well-characterized glioma cell-line, U-87 MG [19] (Fig. 1c). We identified 474 genes that were upregulated in LD^+^ vs LD^−^ GBM cells and expressed in patient samples, out of which 430 genes were also increased in TC^LD+^ vs TC^LD-^ GBM tumor areas (Fig. 1d). Gene ontology (GO) annotation of the 430 commonly upregulated genes revealed the activation of several pathways related to intrinsic cellular biological processes in TC^LD+^, but also to paracrine pro-tumorigenic processes, most notably angiogenesis and exosomes (Fig. 1e and f). Also, components of ER, caveolae, and membrane rafts involved in lipid metabolism and LD formation were significantly enriched (Fig. 1f). Among the commonly upregulated LD^+^ associated genes in GBM cells and patient tumors, we found several hypoxia-related, key effectors of angiogenesis, macrophage recruitment and stromal remodeling in cancer (e.g. *VEGF-A, TGF-β1, IL1β, ADM, IGFB3, LOX, CA9, THBS1, PLODs, ID2*) (Fig. 1g). To specifically elucidate how the hypoxic response may be modulated by LD loading, we next filtered for hypoxia-induced genes (Additional file 1A; Hypoxia vs Normoxia) that were further induced under LD permissive conditions (Additional file 1B; Hypoxia + LDL vs Hypoxia). This identified 22 hypoxia induced genes that were further increased by LD loading in vitro and in TC^LD+^ vs TC^LD-^ areas from patient tumors (Additional file 1C). Given that aggressive tumors, including GBM, follow dynamic cycles of hypoxia and reoxygenation as a result of variations in perfusion [20], we next filtered for LD-induced genes after periods of short (6 h) and long-term (48 h) reoxygenation (Additional file 2A and B). The results suggested that LD-dependent potentiation of the hypoxic response was largely maintained also in the post-hypoxic phase (Additional file 2C and D).

### Lipid loading augments hypoxia mediated secretion of pro-tumorigenic factors

**Fig. 2:**
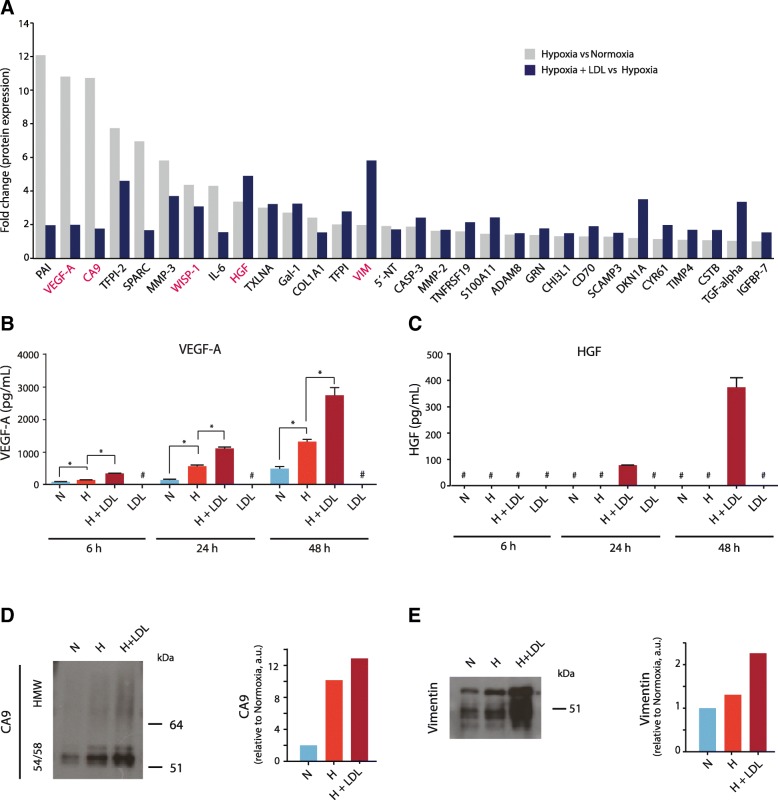
Lipid loading augments hypoxia mediated secretion of pro-tumorigenic factors. **a**, Ultrasensitive proximity extension assay (PEA) profiling of the secreted proteome of GBM cells (U-87 MG) grown in serum free medium for 48 h at normoxic and hypoxic (1% O_2_) conditions with no addition of extracellular lipid or in hypoxia with extracellular lipid (LDL, 50 μg/ml) (*N* = 3 independent experiments). Shown are protein identities increased by at least 1.5-fold (linear) in hypoxia vs normoxia (light gray bars). Dark blue bars show fold increase by lipid loading in hypoxic cells (Hypoxia + LDL vs Hypoxia). ELISA validation of VEGF-A (pg/ml) (**b**) and HGF (pg/ml) (**c**) in the secretome of GBM cells grown under the same conditions as in (**a**) for 6, 24 and 48 h. # = not detected. Western blot analysis of CA9 (**d**) and vimentin (**e**) levels in the secretome of GBM cells grown under the same conditions as in (**a**). Shown are representative blots (left panels) and quantifications (right panels) from at least three independent experiments. HMW: High molecular weight variant of CA9 substituted with glycosaminoglycan

The above findings prompted further elucidation of how extracellular lipid recruitment and LD formation could be mechanistically and functionally linked to paracrine signaling as an essential part of the hypoxic response. We next employed an ultrasensitive immunoassay based on proximity extension assay (PEA) technology to profile the secreted levels of 184 proteins implicated in cancer-stromal cell crosstalk [16–18]. In support of the validity of the assay, several proteins encoded by hypoxia-regulated genes were substantially increased in the hypoxic as compared with the normoxic GBM cell secretome (Fig. 2a, light gray bars). Interestingly, lipid loading was found to augment the hypoxic induction of proteins with key roles in angiogenesis and macrophage recruitment, most importantly VEGF-A (approximately 2-fold) and HGF (approximately 5-fold) (Fig. 2a, dark blue bars). We identified vimentin, i.e. an intermediate filament protein linked to epithelial-to-mesenchymal transition and LD formation [21] as the most highly induced in lipid loading conditions (almost 6-fold as compared with hypoxia only). Several of these responses were maintained also in the reoxygenation phase (Additional file 3A and B). PEA profiling experiments were corroborated by quantitative ELISA, showing that lipid loading significantly amplified the hypoxic induction of VEGF-A (Fig. 2b). This effect was seen already at 6 h (2.6-fold) and was maintained at 24 h and 48 h (2-fold) of lipid loading (Fig. 2b) and was dependent on the concentration of extracellular lipid (Additional file 3C). Remarkably, ELISA analysis showed detectable HGF levels only under conditions of lipid loading, both during 24 and 48 h of hypoxia (Fig. 2c), and also this effect was dependent on extracellular lipid concentration (Additional file 3D). As a control, LDL per se was negative for VEGF-A (Fig. 2b) and HGF (Fig. 2c). Additional proteins of interest were validated by immunoblotting, showing that lipid loading potentiated the hypoxic induction of CA9 and vimentin (Fig. 2d and e). To explore how these results may be reflected by the circulating secretome of GBM patients, we next performed PEA immunoprofiling of plasma from a GBM patient cohort (*N* = 68) as well as in healthy control subjects (*N* = 16) (Additional file 4A). We found significantly increased levels of VEGF-A, HGF, and vimentin in primary GBM (pre-surgery) as compared with healthy controls, and higher levels of these proteins associated with worse patient outcome (Additional file 4B-D).

**Fig. 3:**
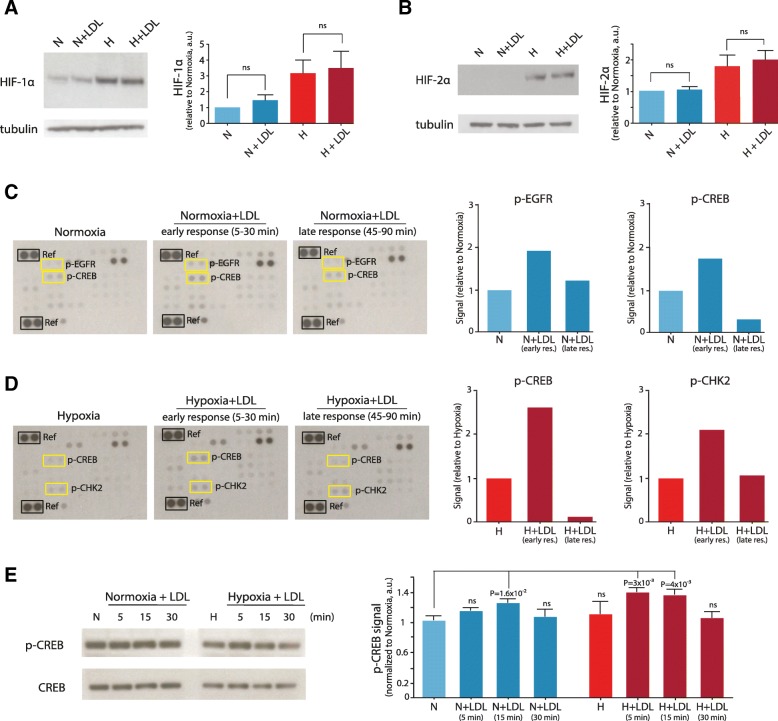
Effects of lipid loading on HIFs and kinase phosphorylation. Western blot analysis of HIF-1α (**a**) and HIF-2 α (**b**) expression in lysates of GBM cells (U-87 MG) grown at normoxic (N) or hypoxic (H) conditions for 24 h in the absence or presence of extracellular lipid (+LDL). Shown are representative blots (left panels) and quantifications (right panels) from three independent experiments. Data are presented as the mean fold of normoxic cells ± SD. Phosphokinase antibody array analysis of lysates from normoxic (**c**) and hypoxic (**d**) GBM cells following no treatment or treatment with extracellular lipid (+LDL) short-term (5, 15, and 30 min) or long-term (45, 60, and 90 min). Shown are representative blots (left panels) and quantifications (right panels) for p-EGFR, p-CREB and p-CHK2. **e**, Western blot p-CREB and total CREB analysis of lysates from normoxic and hypoxic GBM cells following no treatment or treatment with extracellular lipid (+LDL) for the indicated time periods. Shown is a representative blot (left panel) and quantification (right panel) from three independent experiments. Quantification of the protein bands was performed by densitometry using ImageJ, and data were expressed as the ratio between p-CREB to total CREB, and then normalized to normoxic conditions without LDL (CTRL), and are presented as the mean fold hypoxia vs normoxia ± SD

Lipid loading-dependent amplification of the hypoxic response appeared independent on hypoxia inducible factors (HIFs), which serve as transcriptional master regulators of the hypoxic signaling program [22, 23], as lipid loading per se had no apparent effect on HIF-1α and HIF-2α protein levels, neither in normoxia nor in hypoxia (Fig. 3a and b). However, using an antibody array for phosphokinase activation, we found increased phosphorylation of cyclic AMP-response-element binding protein (CREB) (Fig. 3c and d), which has been widely implicated in stress adaptation, and transcriptional induction of VEGF-A through cooperation with HIF-1α [24–27]. Corroborating immunoblotting experiments showed that lipid loading transiently increased the phosphorylated fraction of CREB preferentially in hypoxic conditions (Fig. 3e). Together, these results indicate that lipid loading can amplify the hypoxic response in GBM cells and patient tumors, apparently through mechanisms that do not directly depend on increased HIF stabilization.

### Tumor cell lipid loading promotes paracrine activation of vascular cells and macrophages

**Fig. 4:**
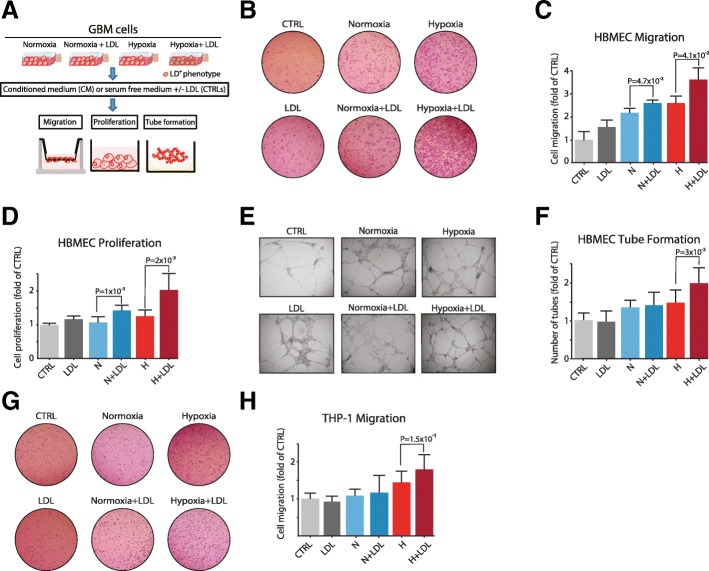
Tumor cell lipid loading promotes paracrine activation of vascular cells and macrophages. **a**, Schematic overview of functional assays with conditioned medium (CM) isolated from GBM cells (U-87 MG) grown in serum-free (SF) medium without and with extracellular lipid (+LDL) in normoxia or hypoxia. **b** and **c**, HBMECs were assessed for transwell migration over a period of 6 h towards SF medium (CTRL) and the various GBM CM variants described in (**a**). LDL = SF medium supplemented with LDL. Shown are representative images of migrated ECs (**b**) and quantification (**c**). **d**, HBMECs were cultured for 72 h at the same conditions as described in (**b**) and assessed for cell proliferation. **e** and **f**, HBMECs were cultured for 24 h at the same conditions as in (**b**) and then allowed to form tube structures on Matrigel for 20 h. Shown are representative images of EC tubes from the indicated treatments (**e**) and quantification of number of tubes (**f**). **g** and **h**, Human-derived monocytes differentiated into macrophages (THP-1) were assessed for transwell migration over a period of 6 h towards the same media conditions as described in (**b**). Shown are representative images of migrated THP-1 cells (**g**) and quantification (**h**). **b-h** Data are presented as the mean fold of untreated (CTRL) ± SD from at least three independent experiments

We next investigated how increased secretion of pro-malignant factors by lipid loading may directly regulate the functional crosstalk with stromal cell components of the GBM tumor microenvironment (Fig. 4a). Based on patient GBM and tumor cell profiling data (Figs. 1 and 2), we initially focused on how the secretome from GBM cells grown under LD permissive (hypoxia + LDL) as compared with non-permissive (normoxia, normoxia + LDL, or hypoxia only) conditions regulates endothelial cell (EC) activity. Interestingly, human brain microvascular EC (HBMEC) migration towards conditioned medium (CM) from hypoxic lipid loaded cells was significantly increased by approximately 1.7-fold and 1.4-fold as compared with CM from normoxic and hypoxic GBM cells, respectively (Fig. 4b and c). The effect on HBMEC cell proliferation was even greater, showing an almost 2-fold induction by lipid loading (Fig. 4d). We found similar effects on HBMECs with the different CM variants derived from GL261 cells, i.e. a widely used mouse high grade glioma model [28, 29] (Additional file 5A and B). Also, CM from lipid loaded GBM cells was shown to significantly potentiate HBMEC tube formation (Fig. 4e and f). Moreover, we found that CM from lipid loaded cells as compared with normoxic cells was significantly more potent at stimulating macrophage migration (Fig. 4g and h). Similar results were obtained in experiments with CM derived from GL261 cells (Additional file 5C). Notably, LDL supplementation per se (LDL) had no direct effect on HBMECs or macrophages (dark grey bars in Fig. 4c, d, f, h and Additional file 5A-C). Together, these results suggest that extracellular lipid loading can amplify hypoxia mediated, paracrine activation of ECs and macrophages.

### Lipid loaded tumor cells foster increased angiogenesis and macrophage infiltration in glioma

**Fig. 5:**
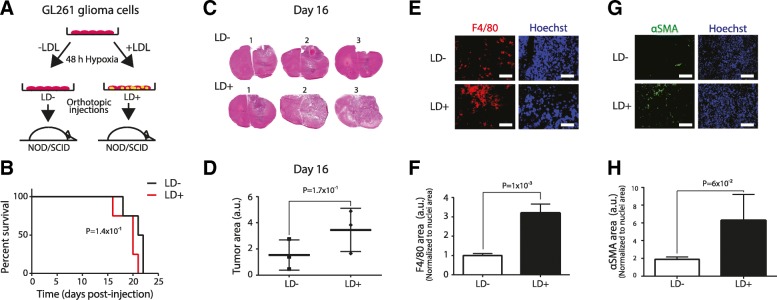
Lipid loading in hypoxic tumor cells promotes angiogenesis and macrophage infiltration in glioma. **a**, GL261 mouse GBM cells were pre-incubated in hypoxia without (LD^−^) or with extracellular lipid (LD^+^) for 48 h, and then injected (50.000 cells/animal) into the brains of NOD/SCID mice. **b**, Kaplan-Meier plot shows survival in LD^−^ and LD^+^ groups (*N* = 5 per group; log-rank test, *P* = 0.141). **c**, H&E staining of brains from the LD^+^ and LD^−^ groups at day 16 post-injection and quantification (**d**) of the largest tumor area (shown are sections from 3 representative animals from each group). **e**, Immunofluorescence staining for the macrophage marker F4/80 (red) and Hoechst nuclei (blue) on frozen tumor sections at day 16, and quantification (**f**) relative to nuclei area (from 3 representative animals per group, 18 fields per animal). Scale bar, 150 μm. **g**, αSMA (green) and Hoechst nuclei (blue) staining at day 16 and quantification (**h**) relative to nuclei area (from 3 representative animals per group, 9 fields per animal). Scale bar, 150 μm

To explore how these findings may be translated to the in vivo setting, we initially employed GL261 cells for orthotopic injection of cells pre-conditioned in hypoxia without (LD negative, LD^−^) and with LDL loading (LD positive, LD^+^), in immunodeficient NOD/SCID mice (Fig. 5a). In this setting, there was a non-significant decrease in animal survival in the LD^+^ as compared with the LD^−^ groups (Fig. 5b; *P* = 0.141) and tumor area (Fig. 5c and d; *P* = 0.175). Interestingly, immunofluorescence mapping of the tumor microenvironment revealed a significantly increased infiltration of tumor associated macrophages (TAM) (> 3-fold; *P* = 0.001; Fig. 5e and f), and there was a strong trend towards an increased tumor density of αSMA-positive, pericyte like vascular cells in the LD^+^ group (> 3-fold; *P* = 0.06; Fig. 5g and h). Together, these data suggest that GBM cell lipid loading confers an increased angiogenic response and TAM infiltration to gliomas grown in immunodeficient mice.

### Hyperlipidemia promotes macrophage infiltration, angiogenesis and GBM aggressiveness

**Fig. 6:**
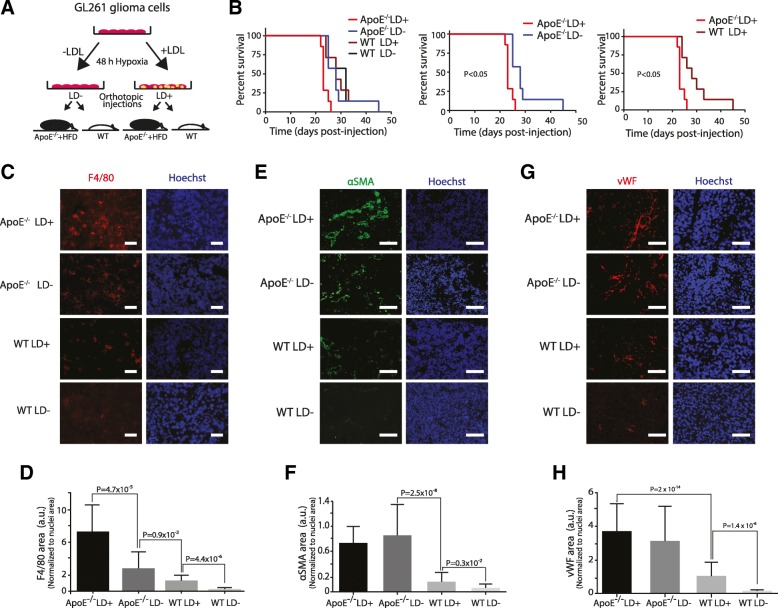
Additive effects of tumor cell lipid loading and systemic lipids on angiogenesis, macrophage infiltration, and survival in glioma. **a**, GL261 mouse GBM cells were pre-incubated in hypoxia without (LD^−^) or with extracellular lipid (LD^+^) for 48 h, and then injected (100.000 cells/animal) into the brains of C57BL/6 mice on chow diet (WT) or hyperlipidemic ApoE^−/−^ mice on high fat diet (HFD). **b**, Kaplan-Meier survival curves (*N* = 7 animals per group). Shown are all groups (left panel), or comparing effect of tumor cell lipid preloading in the hyperlipidemic background (mid panel) or the effect of systemic lipid level background in preloaded tumor cells (right panel). **c**, **e**, and **g**, Immunofluorescence staining of F4/80 (red) (**c**), αSMA (green) (**e**) and vWF endothelial cell marker (red) (**g**) and corresponding quantifications relative to nuclei area (**d**, **f**, and **h**) (from 4 representative animals per group, 9–18 fields per animal for each marker). Scale bars, 150 μm

The above findings motivated further studies on the potential regulatory role of systemic lipids in GBM tumor development, specifically, how hyperlipidemia may regulate angiogenesis and macrophage infiltration in immunocompetent animals. We established the GL261 model in syngeneic C57BL/6 mice, either wild-type (WT) on chow diet or Apolipoprotein E knock-out mice (ApoE^−/−^) that develop severe hyperlipidemia after a few weeks on high fat diet (HFD) [30, 31] (Fig. 6a). Intriguingly, in the hyperlipidemic situation of ApoE^−/−^ mice, orthotopic injection of GL261 LD^+^ as compared with GL261 LD^−^ cells resulted in a significantly decreased survival (Fig. 6b). Moreover, ApoE^−/−^ as compared with WT mice exhibited reduced survival when receiving GL261 LD^+^ cells (Fig. 6b), suggesting that combined tumor cell lipid loading and systemic lipid levels has a significant impact on GBM aggressiveness. Consistent with the results from the NOD/SCID model (Fig. 5), TAM infiltration was significantly increased in mice injected with LD^+^ vs LD^−^ cells, both in WT and ApoE^−/−^ mice (Fig. 6c and d). Interestingly, hyperlipidemia was shown to increase TAMs in mice injected both with LD^+^ and LD^−^ tumor cells, indicating that initial GBM cell lipid loading and an increased systemic lipid supply had additive effects on TAM infiltration (Fig. 6c and d). We found similar effects on blood vessel formation, as assessed by staining for αSMA (Fig. 6e and f) and vWF (Fig. 6g and h), although tumor hypervascularization in systemic hyperlipidemia was not further increased by initial tumor cell lipid loading. Taken together, results from immunodeficient and immunocompetent mouse glioma models (Figs. 5 and 6) provide evidence that lipid levels, both locally in the tumor cells and systemically, can promote angiogenesis, TAM infiltration and GBM tumor aggressiveness.

## Discussion

The current work provides new insights into the role of extracellular lipids and the LD phenotype in the regulation of hypoxia mediated tumor development by an increased angiogenic response and macrophage recruitment. We show that the LD phenotype of patient tumors is associated with an enhanced hypoxic response, and that extracellular lipids can augment hypoxic paracrine signaling by increased secretion of key effector molecules involved in shaping the GBM stromal compartment. Our results reveal a reciprocal crosstalk between hypoxia and lipid loading and underscore the potential synergy between autocrine and paracrine mechanisms during metabolic adaptation to tumor stressness. This should motivate future studies focusing on how the lipoprotein uptake machinery may be targeted as an effective therapeutic approach, especially in notoriously hypoxic GBM tumors.

The correlation of angiogenesis and abundance of macrophages with glioma grade and poor GBM patient outcome is well-documented [32, 33]. Here, we found a substantial increase in TAM infiltration and neovascularization in tumors established from LD^+^ GBM cells, and this effect was augmented by an elevated, continuous supply of systemic lipids. Moreover, we show that lipid loading can induce the secretion of major, pro-malignant factors implicated in TAM recruitment and the angiogenic response, i.e. VEGF-A and HGF [34, 35]. Tumor hypervascularization in systemic hyperlipidemia was not further increased by tumor cell LD loading, i.e. the effects of systemic hyperlipidemia per se on tumor angiogenesis may hide the effects of tumor cell LD loading in this setup. Notably, TAM recruitment with LD+ GBM cells was increased not only in the WT group but also in the hyperlipidemic background, suggesting that in this context the additive effect of systemic lipid levels and tumor cell LD loading on animal survival is mediated through increased macrophage infiltration. TAMs may represent more than half of the GBM tumor mass [35], and previous studies in the GL261 mouse glioma model suggested that the relative contribution of peripheral monocytes and macrophages vs resident microglia progressed over time to more than 1/3 of the total TAM population [36]. TAMs thus constitute a mixed cell population that can promote GBM pathology by immunosuppressive actions but also as a major source of angiogenic growth factors, including VEGF [32]. Accordingly, targeting of TAMs through inhibition of CSF1R increased the survival in experimental GBM [37] and may improve the effectiveness of anti-angiogenic therapies [38]. It is conceivable that the hypervascularized phenotype of lipid loaded tumors, as shown here, is the result of pro-angiogenic signaling directly by malignant cells but also secondary to TAM recruitment.

The leaky vasculature of GBM, and other hypoxic tumors, allows the recruitment of systemic lipids, including free fatty acids with mean concentrations of approximately 50–120 mM in plasma and the considerably more abundant cholesteryl esters (approximately 400–750 mM) and triglycerides sequestered in lipoproteins [39]. Tumor tissues readily have access to and metabolize circulating lipids, and LDL was shown to transcytose over the endothelial cell barrier [40]. Also, in conditions where the endothelial barrier integrity is compromised, lipoproteins can have direct access deep into tissues [41]. In favor of this notion, the present study shows that HFD/hyperlipidemia can synergize with tumor cell lipid loading to promote GBM tumor remodeling and aggressiveness, thereby establishing a mechanistic and causal link between lipid accumulation and tumor progression. Obesity is a well-established risk factor in many cancers, including breast and colon cancer, as well as clear cell renal cell carcinoma [42]. How obesity is affecting patients with high-grade glioma is, however, still unclear, but a correlation has been suggested between obesity and an enhanced risk of brain tumors [43, 44–46].

## Conclusions

Our findings high-light the role of lipid metabolism in cancer by revealing an association between cancer cell lipid loading and increased hypoxic stress adaptive paracrine signaling, resulting in augmented cross-activation of major inhabitants of the stressed GBM tumor niche. We conclude that the studied mechanisms represent potentially targetable modulators of the adaptive metabolic response, which should motivate the identification of new drugs or repurposing of drugs from the cardiovascular field targeted at extracellular lipid internalization and LD formation for the treatment of GBM.

## Additional files

Additional file 1: Lipid loading potentiates the hypoxic response in GBM cells and patient tumors. Shown is hypoxic regulation (Hypoxia vs Normoxia) (A) and further amplification of gene expression by lipid loading (Hypoxia + LDL vs Hypoxia) (B) in GBM (U-87 MG) cells. C, Heatmap of differentially expressed genes in GBM cells and patient tumors. Column 1: Upregulated genes by hypoxia in GBM cells (Hypoxia vs Normoxia). Column 2: Hypoxia-induced genes further induced by lipid loading in GBM cells (Hypoxia + LDL vs Hypoxia). Column 3: Differential expression of genes presented in Column 1 and 2 for patient Tumor CellLD^+^ and Tumor CellLD^−^ (Tumor CellLD^+^ vs Tumor CellLD^−^) areas isolated by laser microdissection. Bar graph (right panel) highlights the relative expression of commonly upregulated genes in Tumor CellLD^+^ vs Tumor CellLD^−^ (gray bars) and Hypoxia + LDL vs Normoxia (black bars). Data (A-C) is presented as log_2_ fold-change (FC). (PDF 36 kb)

Additional file 2: Maintained effect of lipid loading on gene expression in reoxygenated, post-hypoxic conditions. A, GBM cells (U-87 MG) were grown in hypoxia without (LD^−^) and with LDL (LD^+^) and then reoxygenated. Shown are genes induced at least 1.1 log_2_ of fold change (FC) in LD+ vs LD- cells following 6 h of reoxygenation. B, Same analysis as in (A) at 48 h of reoxygenation. C, Expression of the commonly upregulated genes in Tumor CellLD^+^ vs Tumor CellLD^−^ and Hypoxia +LDL vs Hypoxia, as shown in Additional file 1C, in LD^+^ vs LD^−^ cells following 6 h of reoxygenation. In all cases, there is a maintained induction by lipid loading. D, Same analysis as in (C) at 48 h of reoxygenation. (PDF 15 kb)

Additional file 3: Effects of lipid loading on the hypoxic and post-hypoxic GBM cell secretome. A and B, Maintained effect of lipid loading on protein secretion in reoxygenated, post-hypoxic conditions: GBM cells (U-87 MG) were grown in hypoxia without (LD^−^) and with LDL (LD^+^) and then reoxygenated for 6 h (A) and 48 h (B). Shown is linear fold change (FC) of protein levels in LD^+^ vs LD^−^ cells as determined by proximity extension assay. C and D, ELISA quantification of VEGF-A (C) and HGF (D) in the secretome of GBM cells grown in normoxia (N), hypoxia without LDL (H) or in hypoxia with LDL at the indicated concentrations. LDL = LDL only (50 μg/ml). # = Not detected. (PDF 16 kb)

Additional file 4: VEGF-A, HGF, and vimentin are increased in GBM patient plasma and correlate with disease aggressiveness. A, Proximity extension assay (PEA) immunoprofiling of proteins in plasma from GBM patients (*N* = 68) and healthy controls (*N* = 16) shows that several proteins increased by LD loading in vitro (as shown in Fig. 2a) are also increased in GBM patient plasma. Data shown are corrected values (for age and sex) presented as the mean linear fold change. *P ≤ 0.05. B-D, 1-year survival of GBM patients with high or low expression levels of VEGF-A (B), vimentin (VIM) (C) and HGF (D). (PDF 45 kb)

Additional file 5: Tumor cell lipid loading promotes paracrine activation of vascular cells and macrophages. See Fig. 4a for a schematic overview of functional assays with conditioned medium (CM) isolated from GBM cells (U-87 MG or GL261) grown in serum-free (SF) medium without and with extracellular lipid (+LDL) in normoxia (N) or hypoxia (H). A, HBMECs were assessed for transwell migration over a period of 6 h towards SF medium (CTRL) and the various GL261 CM variants, as indicated. LDL = SF medium supplemented with LDL. B, HBMECs were cultured for 72 h at the same conditions as described in (A) and assessed for cell proliferation. C, Human derived monocytes differentiated into macrophages (THP-1) were assessed for transwell migration over a period of 6 h towards the same media conditions as described in (A and B). A-C, Data are presented as the mean fold of untreated (CTRL) ± SD from at least three independent experiments. (PDF 12 kb)

## Abbreviations

ApoE: Apolipoprotein E
aSMA: Alpha smooth muscle actin
CM: Conditioned medium
CREB: Cyclic AMP-response-element binding protein
GBM: Glioblastoma
H: Hypoxia
HBMEC: Human brain endothelial cells
HFD: High fat diet
HIF: Hypoxia inducible factor
LD: Lipid droplet
LDL: Low density lipoprotein
N: Normoxia
PEA: Proximity extension assay
PFA: Paraformaldehyde
RT: Room temperature
SF: Serum free
TAM: Tumor associated macrophage
TC: Tumor cell
vWF: Von Willebrandt factor
WT: Wild-type

## References

[1] Louis DN, Perry A, Reifenberger G, von Deimling A, Figarella-Branger D, Cavenee WK (2016). The 2016 World Health Organization classification of tumors of the central nervous system: a summary. Acta Neuropathol.

[2] Omuro A (2013). Glioblastoma and other malignant gliomas. JAMA.

[3] Quail DF, Joyce JA (2013). Microenvironmental regulation of tumor progression and metastasis. Nat Med.

[4] Schulze A, Harris AL (2012). How cancer metabolism is tuned for proliferation and vulnerable to disruption. Nature.

[5] Strickland M, Stoll EA (2017). Metabolic reprogramming in glioma. Front Cell Dev Biol.

[6] Lin H, Patel S, Affeck VS, Wilson I, Turnbull DM, Joshi AR (2017). Fatty acid oxidation is required for the respiration and proliferation of malignant glioma cells. Neuro-Oncology.

[7] Delikatny EJ, Chawla S, Leung DJ, Poptani H (2011). MR-visible lipids and the tumor microenvironment. NMR Biomed.

[8] Menard JA, Christianson HC, Kucharzewska P, Bourseau-Guilmain E, Svensson KJ, Lindqvist E (2016). Metastasis stimulation by hypoxia and acidosis-induced extracellular lipid uptake is mediated by proteoglycan-dependent endocytosis. Cancer Res.

[9] Ackerman D, Simon MC (2014). Hypoxia, lipids, and cancer: surviving the harsh tumor microenvironment. Trends Cell Biol.

[10] Ackerman D, Tumanov S, Qiu B, Michalopoulou E, Spata M, Azzam A (2018). Triglycerides promote lipid homeostasis during hypoxic stress by balancing fatty acid saturation. Cell Rep.

[11] Furuta E, Pai SK, Zhan R, Bandyopadhyay S, Watabe M, Mo YY (2008). Fatty acid synthase gene is up-regulated by hypoxia via activation of Akt and sterol regulatory element binding Protein-1. Cancer Res.

[12] Bensaad K, Favaro E, Lewis CA, Peck B, Lord S, Collins JM (2014). Fatty acid uptake and lipid storage induced by HIF-1α contribute to cell growth and survival after hypoxia-Reoxygenation. Cell Rep.

[13] Hoang-Minh LB, Siebzehnrubl FA, Yang C, Suzuki-Hatano S, Dajac K, Loche T (2018). Infiltrative and drug-resistant slow-cycling cells support metabolic heterogeneity in glioblastoma. EMBO J.

[14] Negendank WG, Sauter R, Brown TR, Evelhoch JL, Falini A, Gotsis ED (1996). Proton magnetic resonance spectroscopy in patients with glial tumors: a multicenter study. J Neurosurg.

[15] Yue S, Li J, Lee S-Y, Lee HJ, Shao T, Song B (2014). Cholesteryl Ester accumulation induced by PTEN loss and PI3K/AKT activation underlies human prostate Cancer aggressiveness. Cell Metab.

[16] Indira Chandran Vineesh, Welinder Charlotte, Månsson Ann-Sofie, Offer Svenja, Freyhult Eva, Pernemalm Maria, Lund Sigrid M., Pedersen Shona, Lehtiö Janne, Marko-Varga Gyorgy, Johansson Maria C., Englund Elisabet, Sundgren Pia C., Belting Mattias (2019). Ultrasensitive Immunoprofiling of Plasma Extracellular Vesicles Identifies Syndecan-1 as a Potential Tool for Minimally Invasive Diagnosis of Glioma. Clinical Cancer Research.

[17] Enroth S, Johansson Å, Enroth SB, Gyllensten U (2014). Strong effects of genetic and lifestyle factors on biomarker variation and use of personalized cutoffs. Nat Commun.

[18] Larssen P, Wik L, Czarnewski P, Eldh M, Löf L, Ronquist KG (2017). Tracing cellular origin of human exosomes using multiplex proximity extension assays. Mol Cell Proteomics.

[19] Clark MJ, Homer N, O'Connor BD, Chen Z, Eskin A, Lee H (2010). U87MG decoded: the genomic sequence of a cytogenetically aberrant human Cancer cell line. PLoS Genet.

[20] Michiels C, Tellier C, Feron O (2016). Cycling hypoxia: a key feature of the tumor microenvironment. Biochim Biophys Acta.

[21] Londos C, Brasaemle DL, Schultz CJ, Segrest JP, Kimmel AR (1999). Perilipins, ADRP, and other proteins that associate with intracellular neutral lipid droplets in animal cells. Semin Cell Dev Biol.

[22] Kaelin WG (2008). The von Hippel–Lindau tumour suppressor protein: O2 sensing and cancer. Nat Rev Cancer.

[23] Semenza GL (2009). Regulation of cancer cell metabolism by hypoxia-inducible factor 1. Semin Cancer Biol.

[24] Mayr B, Montminy M (2001). Transcriptional regulation by the phosphorylation-dependent factor creb. Nat Rev Mol Cell Biol.

[25] Barresi V, Mondello S, Branca G, Rajan TS, Vitarelli E, Tuccari G (2015). p-CREB expression in human gliomas: potential use in the differential diagnosis between astrocytoma and oligodendroglioma. Hum Pathol.

[26] Chhipa RR, Fan Q, Anderson J, Muraleedharan R, Huang Y, Ciraolo G (2018). AMP kinase promotes glioblastoma bioenergetics and tumour growth. Nat Cell Biol.

[27] Wu D, Zhau HE, Huang WC, Iqbal S, Habib FK, Sartor O (2007). cAMP-responsive element-binding protein regulates vascular endothelial growth factor expression: implication in human prostate cancer bone metastasis. Oncogene.

[28] Ausman JI, Shapiro WR, Rall DP (1970). Studies on the chemotherapy of experimental brain tumors: development of an experimental model. Cancer Res.

[29] Chen Z, Feng X, Herting CJ, Garcia VA, Nie K, Pong WW (2017). Cellular and molecular identity of tumor-associated macrophages in glioblastoma. Cancer Res.

[30] Véniant MM, Withycombe S, Young SG (2001). Lipoprotein size and atherosclerosis susceptibility in Apoe(−/−) and Ldlr(−/−) mice. Arterioscler Thromb Vasc Biol.

[31] Piedrahita JA, Zhang SH, Hagaman JR, Oliver PM, Maeda N (1992). Generation of mice carrying a mutant apolipoprotein E gene inactivated by gene targeting in embryonic stem cells. Proc Natl Acad Sci U S A.

[32] Quail DF, Joyce JA (2017). The microenvironmental landscape of brain tumors. Cancer Cell.

[33] Gabrusiewicz K, Rodriguez B, Wei J, Hashimoto Y, Healy LM, Maiti SN (2016). Glioblastoma-infiltrated innate immune cells resemble M0 macrophage phenotype. JCI Insight.

[34] Hambardzumyan D, Gutmann DH, Kettenmann H (2016). The role of microglia and macrophages in glioma maintenance and progression. Nat Neurosci.

[35] Broekman ML, Maas SLN, Abels ER, Mempel TR, Krichevsky AM, Breakefield XO (2018). Multidimensional communication in the microenvirons of glioblastoma. Nat Rev Neurol.

[36] Müller A, Brandenburg S, Turkowski K, Müller S, Vajkoczy P (2015). Resident microglia, and not peripheral macrophages, are the main source of brain tumor mononuclear cells. Int J Cancer.

[37] Pyonteck SM, Akkari L, Schuhmacher AJ, Bowman RL, Sevenich L, Quail DF (2013). CSF-1R inhibition alters macrophage polarization and blocks glioma progression. Nat Med.

[38] Mantovani A, Marchesi F, Malesci A, Laghi L, Allavena P (2017). Tumour-associated macrophages as treatment targets in oncology. Nat Rev Clin Oncol.

[39] Psychogios N, Hau DD, Peng J, Guo AC, Mandal R, Bouatra S (2011). The human serum metabolome. PLoS One.

[40] Von Eckardstein A, Rohrer L (2009). Transendothelial lipoprotein transport and regulation of endothelial permeability and integrity by lipoproteins. Curr Opin Lipidol.

[41] Tabas I, García-Cardeña G, Owens GK (2015). Recent insights into the cellular biology of atherosclerosis. J Cell Biol.

[42] Lauby-Secretan B, Scoccianti C, Loomis D, Grosse Y, Bianchini F, Straif K (2016). Body fatness and Cancer — viewpoint of the IARC working group. N Engl J Med.

[43] Niedermaier T, Behrens G, Schmid D, Schlecht I, Fischer B, Leitzmann MF (2015). Body mass index, physical activity, and risk of adult meningioma and glioma. Neurology.

[44] Zhang D, Chen J, Wang J, Gong S, Jin H, Sheng P (2016). Body mass index and risk of brain tumors: a systematic review and dose–response meta-analysis. Eur J Clin Nutr.

[45] Benson VS, Pirie K, Green J, Casabonne D, Beral V (2008). Lifestyle factors and primary glioma and meningioma tumours in the million women study cohort. Br J Cancer.

[46] Moore SC, Rajaraman P, Dubrow R, Darefsky AS, Koebnick C, Hollenbeck A (2009). Height, body mass index, and physical activity in relation to glioma risk. Cancer Res.

