# Supplementary figures and images for: Extracellular lipid loading augments hypoxic paracrine signaling and promotes glioma angiogenesis and macrophage infiltration
Source: J Exp Clin Cancer Res. 2019 Jun 7;38:241. doi: 10.1186/s13046-019-1228-6 (PMC6556032; doi:10.1186/s13046-019-1228-6)

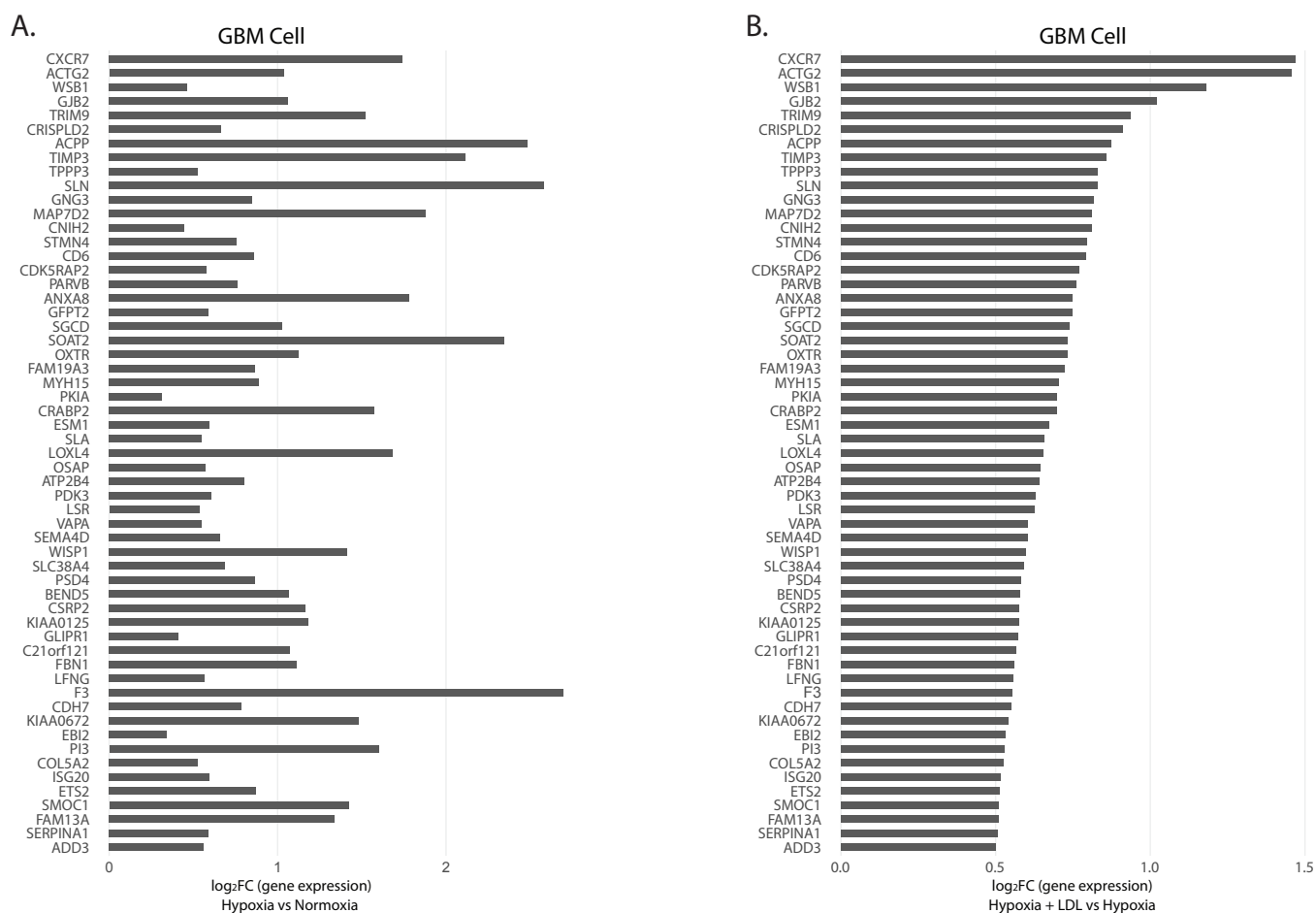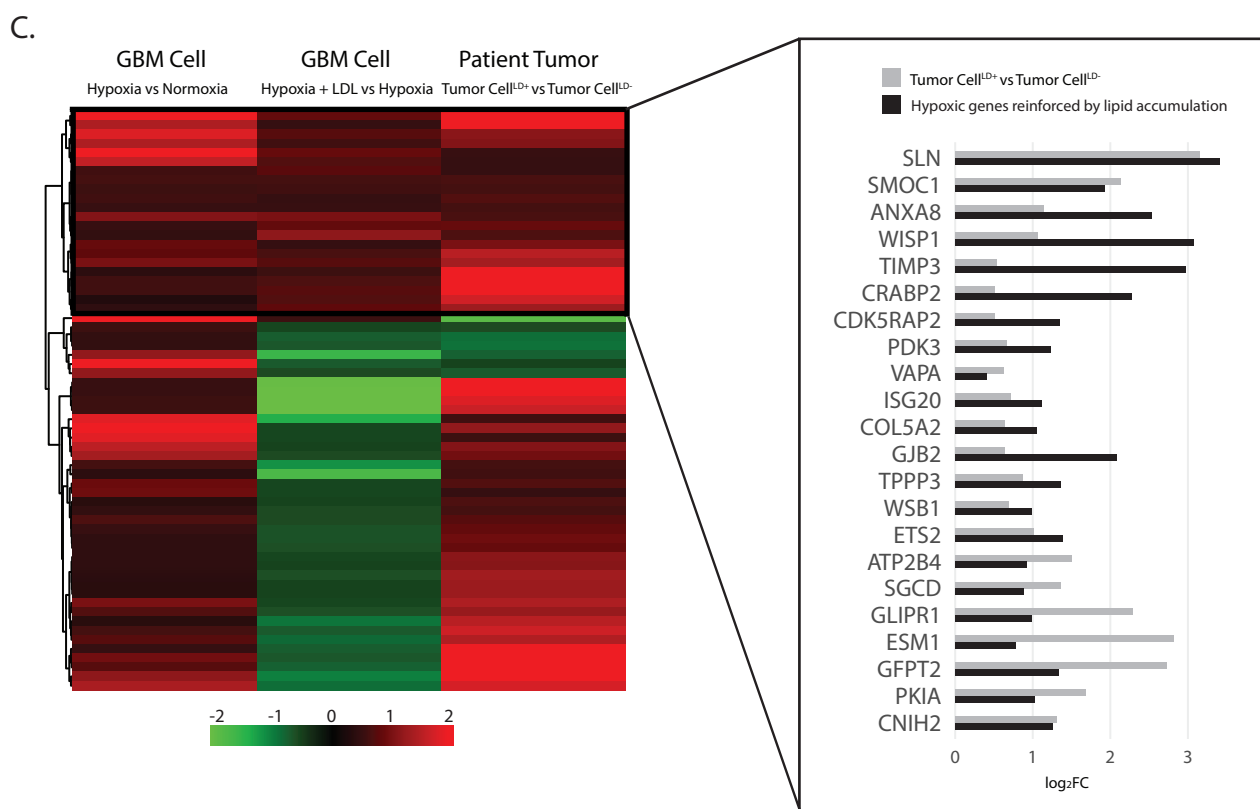

Supplement: Supplementary file 1 — Lipid loading potentiates the hypoxic response in GBM cells and patient tumors. Shown is hypoxic regulation (Hypoxia vs Normoxia) (A) and further amplification of gene expression by lipid loading (Hypoxia + LDL vs Hypoxia) (B) in GBM (U-87 MG) cells. C, Heatmap of differentially expressed genes in GBM cells and patient tumors. Column 1: Upregulated genes by hypoxia in GBM cells (Hypoxia vs Normoxia). Column 2: Hypoxia-induced genes further induced by lipid loading in GBM cells (Hypoxia + LDL vs Hypoxia). Column 3: Differential expression of genes presented in Column 1 and 2 for patient Tumor CellLD+ and Tumor CellLD− (Tumor CellLD+ vs Tumor CellLD−) areas isolated by laser microdissection. Bar graph (right panel) highlights the relative expression of commonly upregulated genes in Tumor CellLD+ vs Tumor CellLD− (gray bars) and Hypoxia + LDL vs Normoxia (black bars). Data (A-C) is presented as log2 fold-change (FC). (PDF 36 kb) [file 13046_2019_1228_MOESM1_ESM.pdf]

A.

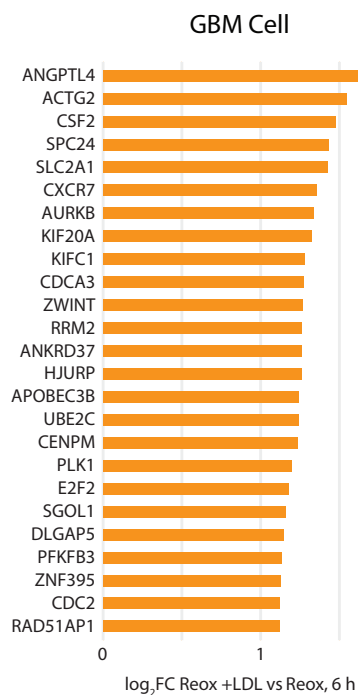

B.

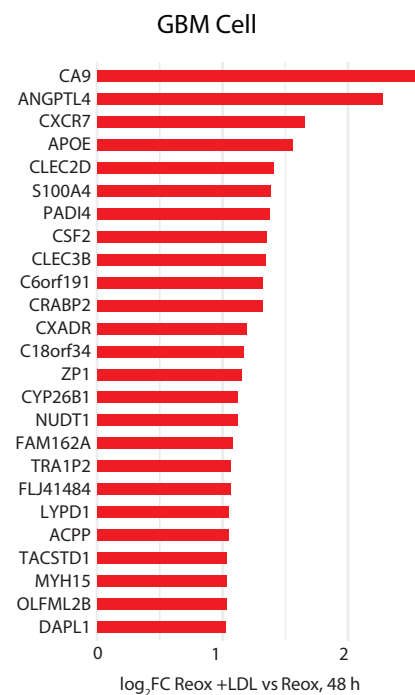

C.

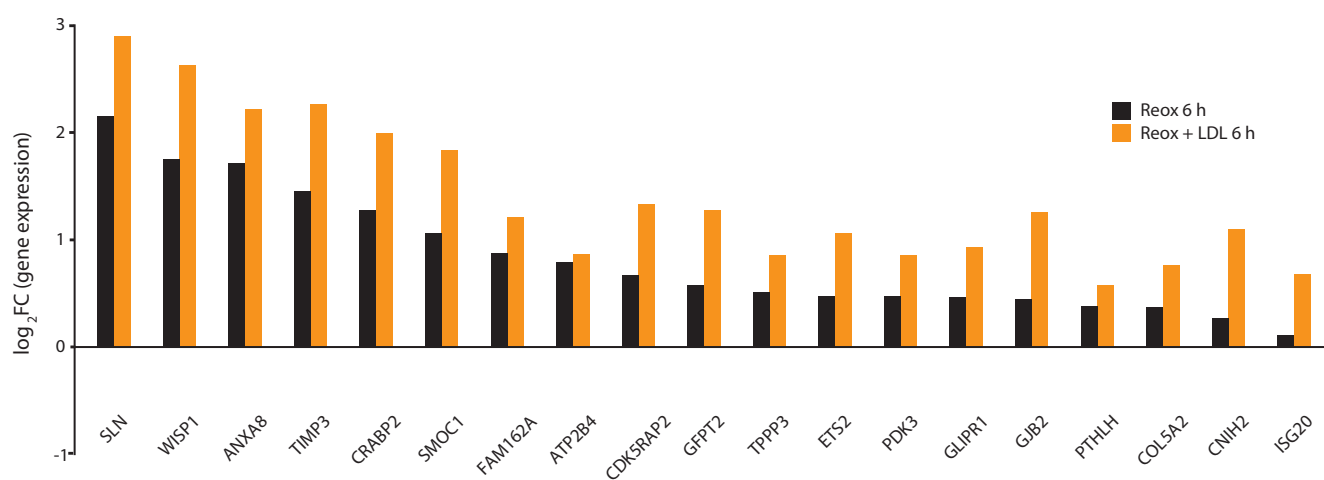

D.

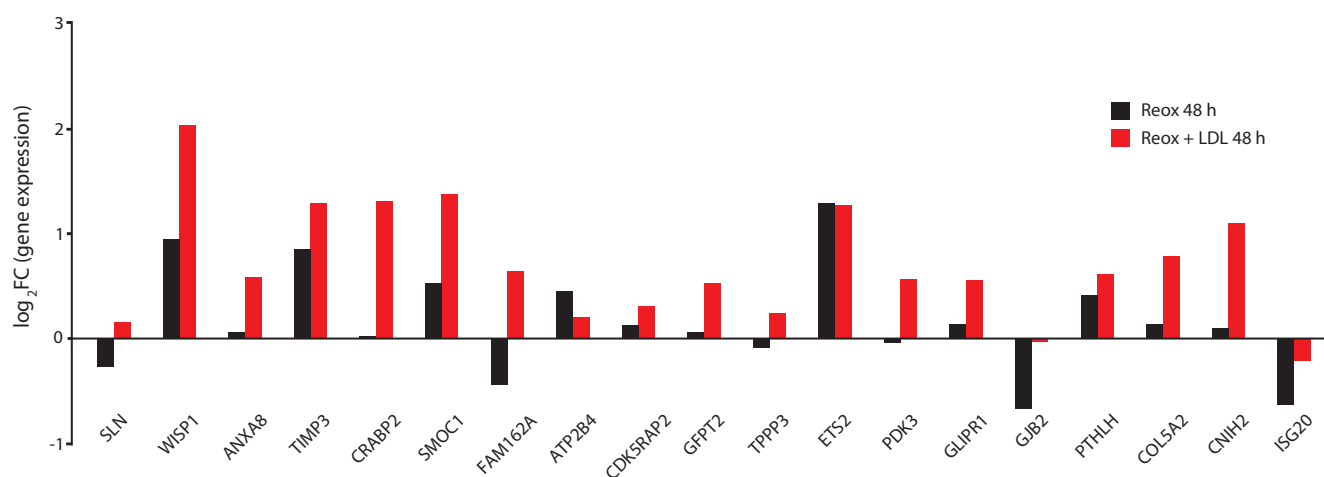

Supplement: Supplementary file 2 — Maintained effect of lipid loading on gene expression in reoxygenated, post-hypoxic conditions. A, GBM cells (U-87 MG) were grown in hypoxia without (LD−) and with LDL (LD+) and then reoxygenated. Shown are genes induced at least 1.1 log2 of fold change (FC) in LD+ vs LD- cells following 6 h of reoxygenation. B, Same analysis as in (A) at 48 h of reoxygenation. C, Expression of the commonly upregulated genes in Tumor CellLD+ vs Tumor CellLD− and Hypoxia +LDL vs Hypoxia, as shown in Additional file 1C, in LD+ vs LD− cells following 6 h of reoxygenation. In all cases, there is a maintained induction by lipid loading. D, Same analysis as in (C) at 48 h of reoxygenation. (PDF 15 kb) [file 13046_2019_1228_MOESM2_ESM.pdf]

A.

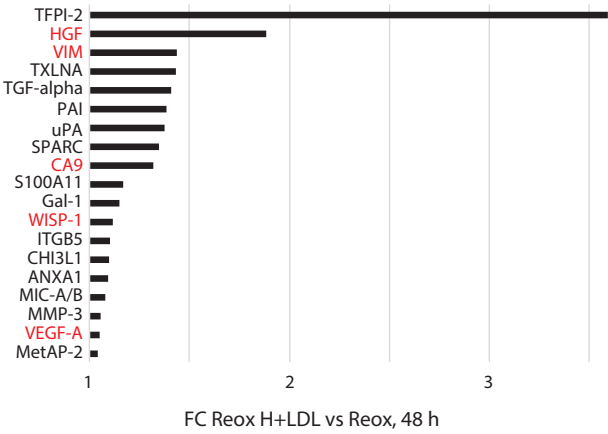

B.

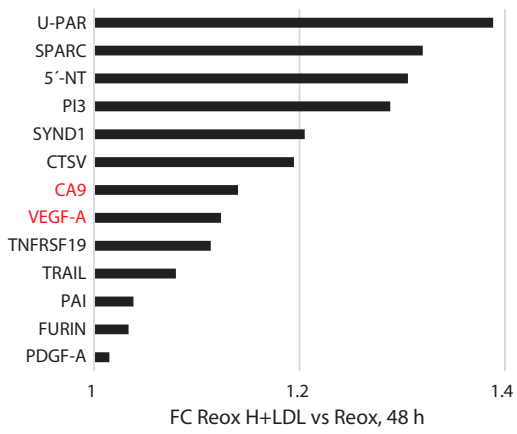

C.

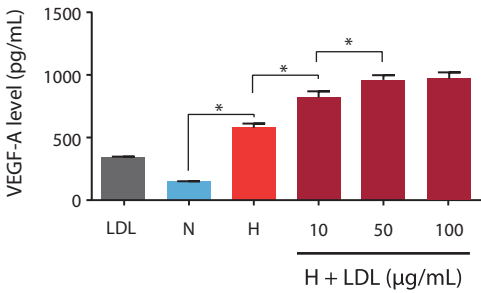

D.

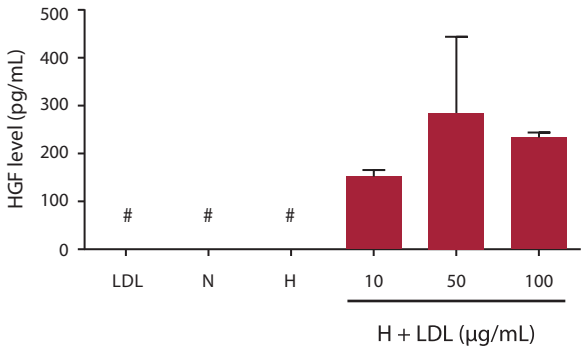

Supplement: Supplementary file 3 — Effects of lipid loading on the hypoxic and post-hypoxic GBM cell secretome. A and B, Maintained effect of lipid loading on protein secretion in reoxygenated, post-hypoxic conditions: GBM cells (U-87 MG) were grown in hypoxia without (LD−) and with LDL (LD+) and then reoxygenated for 6 h (A) and 48 h (B). Shown is linear fold change (FC) of protein levels in LD+ vs LD− cells as determined by proximity extension assay. C and D, ELISA quantification of VEGF-A (C) and HGF (D) in the secretome of GBM cells grown in normoxia (N), hypoxia without LDL (H) or in hypoxia with LDL at the indicated concentrations. LDL = LDL only (50 μg/ml). # = Not detected. (PDF 16 kb) [file 13046_2019_1228_MOESM3_ESM.pdf]

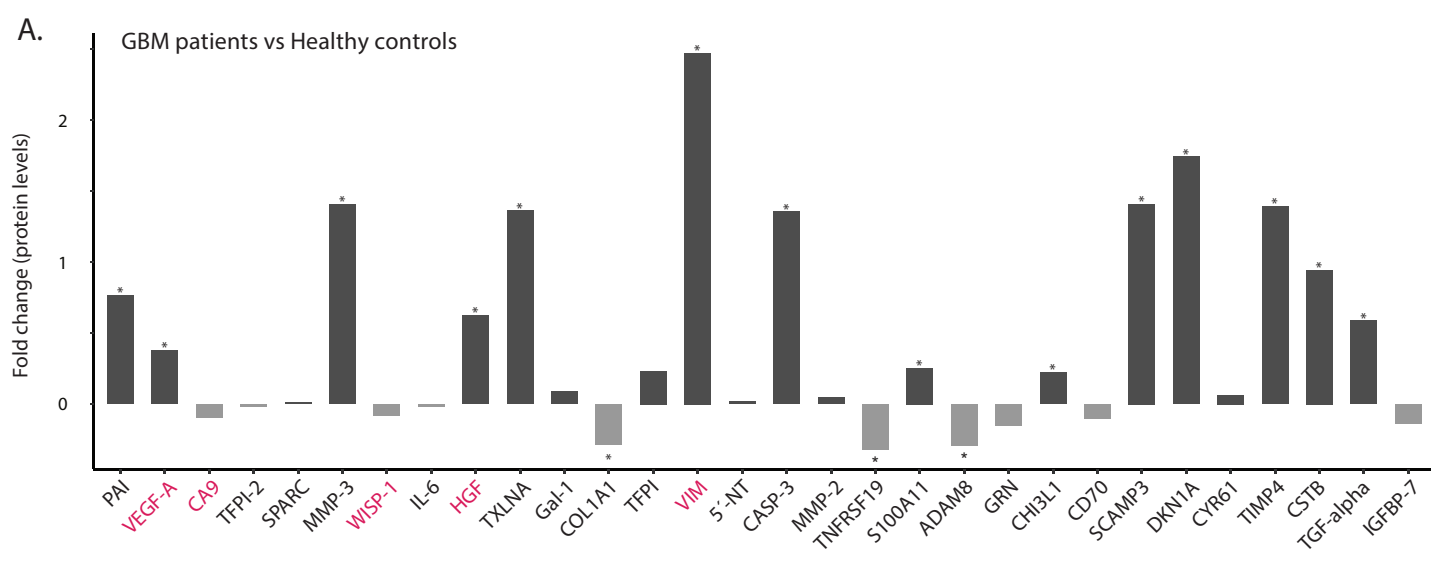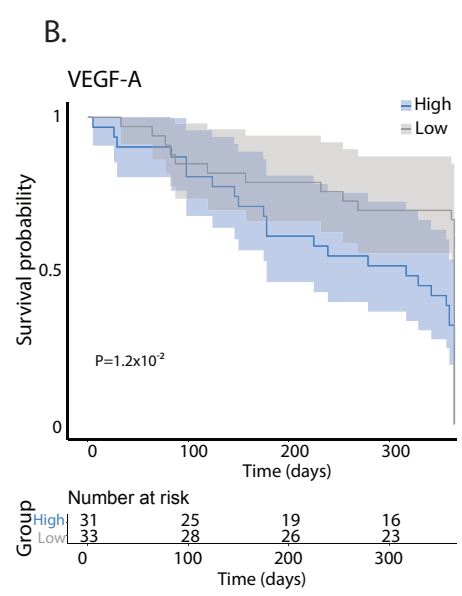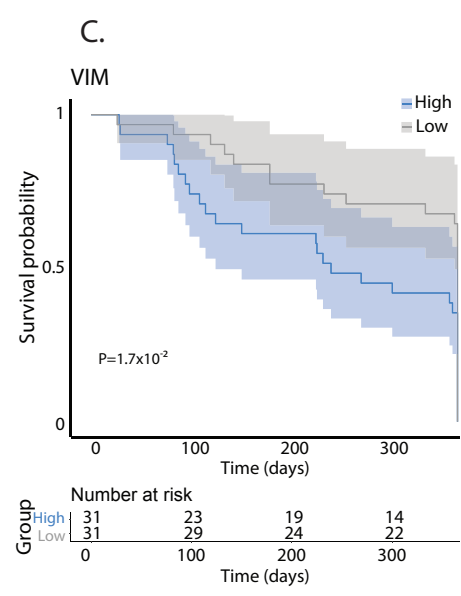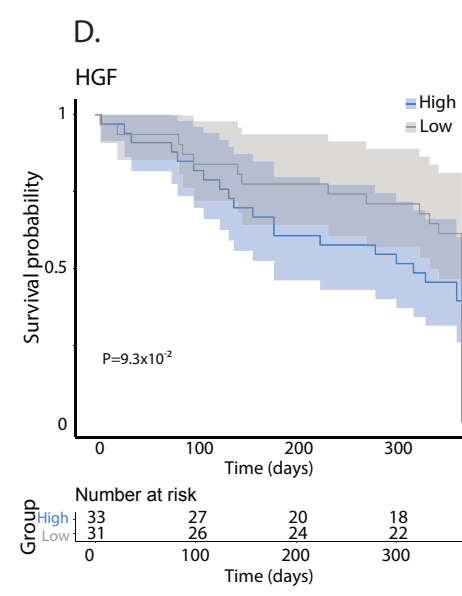

Supplement: Supplementary file 4 — VEGF-A, HGF, and vimentin are increased in GBM patient plasma and correlate with disease aggressiveness. A, Proximity extension assay (PEA) immunoprofiling of proteins in plasma from GBM patients (N = 68) and healthy controls (N = 16) shows that several proteins increased by LD loading in vitro (as shown in Fig. 2a) are also increased in GBM patient plasma. Data shown are corrected values (for age and sex) presented as the mean linear fold change. *P ≤ 0.05. B-D, 1-year survival of GBM patients with high or low expression levels of VEGF-A (B), vimentin (VIM) (C) and HGF (D). (PDF 45 kb) [file 13046_2019_1228_MOESM4_ESM.pdf]

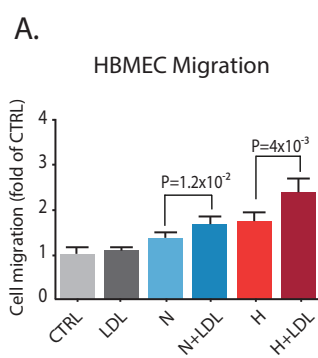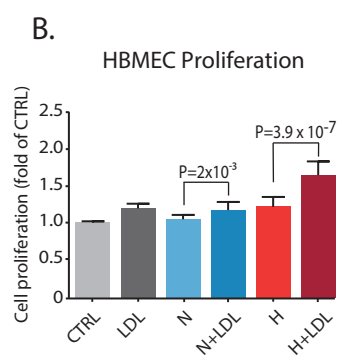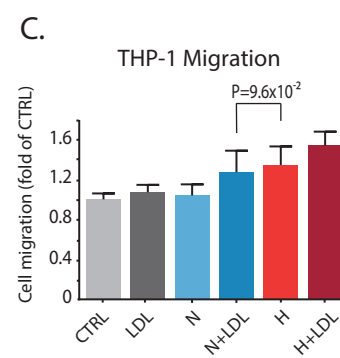

Supplement: Supplementary file 5 — Tumor cell lipid loading promotes paracrine activation of vascular cells and macrophages. See Fig. 4a for a schematic overview of functional assays with conditioned medium (CM) isolated from GBM cells (U-87 MG or GL261) grown in serum-free (SF) medium without and with extracellular lipid (+LDL) in normoxia (N) or hypoxia (H). A, HBMECs were assessed for transwell migration over a period of 6 h towards SF medium (CTRL) and the various GL261 CM variants, as indicated. LDL = SF medium supplemented with LDL. B, HBMECs were cultured for 72 h at the same conditions as described in (A) and assessed for cell proliferation. C, Human derived monocytes differentiated into macrophages (THP-1) were assessed for transwell migration over a period of 6 h towards the same media conditions as described in (A and B). A-C, Data are presented as the mean fold of untreated (CTRL) ± SD from at least three independent experiments. (PDF 12 kb) [file 13046_2019_1228_MOESM5_ESM.pdf]
